# Age and Post-Prandial Variations on Selected Metabolites in Dairy Calves Fed Different Liquid Diets

**DOI:** 10.3390/ani12213063

**Published:** 2022-11-07

**Authors:** Amanda Moelemberg Cezar, Sophia Cattleya Dondé, Cristiane Regina Tomaluski, Ana Paula da Silva, Ariany Faria de Toledo, Marina Gavanski Coelho, Gercino Ferreira Virgínio Júnior, Carla Maris Machado Bittar

**Affiliations:** Department of Animal Science, Luiz de Queiroz College of Agriculture, University of Sao Paulo, Av. Pádua Dias, 11, Piracicaba 13418-900, Brazil

**Keywords:** β-hydroxybutyrate, energetic metabolism, glycemic status

## Abstract

**Simple Summary:**

The aim of this study is to evaluate the age and post-prandial variations in selected metabolites’ concentration during the preweaning period, which may indicate a shift in metabolism, from pre-ruminant to a functional ruminant, according to the liquid diet (whole milk or milk replacer) fed to dairy calves. An additional goal was to evaluate fructosamine concentrations as a replacement for glucose to better understand the glycemic status and possible use of it as a biomarker of rumen development. Plasma glucose concentration did not differ between the liquid diets. The sampling time to study the glycemic status varies with the liquid diet. The best collection time is 1 and 4 hours after being fed whole milk or milk replacer, respectively. Fructosamine was not informative for an indirect evaluation of ruminal development.

**Abstract:**

The aim of this study was to evaluate the age and post-prandial variations in selected metabolite concentration that may indicate a shift in metabolism, from pre- to functional ruminant, according to the liquid diet fed to dairy calves. Sixteen newborn Holstein calves were included in the study in a randomized complete block experimental design. The calves were individually housed and fed 6 L/d with whole milk (WM) or milk replacer (MR). Blood samples were collected weekly at 0 h (before feeding), 1 h, 2 h, 4 h, and 8 h after morning feeding to evaluate glucose, β-hydroxybutyrate (BHB), fructosamine, total protein, and albumin. Calves fed WM had higher performance (*p* < 0.01) than did calves fed MR. The different liquid diets did not affect the average concentrations of plasma glucose. However, BHB was higher for WM-fed calves (*p* < 0.01). The concentration of plasma glucose reached the highest concentration at 1 and 4 hours after feeding WM or MR, respectively. Thus, these would be the most appropriate sampling times to study the glycemic status of calves according to the liquid diet fed. Fructosamine did not prove to be an informative metabolite to understand the shift in metabolism, as a function of rumen development, due to a small reduction as a function of age and a sampling time effect.

## 1. Introduction

The energy metabolism of young dairy calves experiences major changes in the pre-weaning period with the shift from a pre-ruminant to a functional ruminant metabolism [1,2]. As the rumen develops, the main biochemical constituents of energy metabolism, such as glucose, cholesterol, and β-hydroxybutyrate (BHB), significantly change their concentrations [3,4]. This period is characterized by changes in the intestinal absorption of glucose, long-chain fatty acids, and amino acids from milk to initiate the utilization of short-chain fatty acids (SCFAs), ketone bodies, amino acids, and microbial protein from rumen fermentation [1]. Once the calves increase the solid diet intake and rumen fermentation processes, the liquid diet contributes less as the primary source of energy [5]. Metabolism changes occur with increasing ruminal fermentative activity and an antagonistic relationship between glucose and BHB concentrations is observed since glucose concentration decreases and BHB increases [2]. As a result, the preweaning glycemic status and BHB levels are indicators of the transition from a pre-ruminant to a functional ruminant condition.

Although glucose and BHB concentrations are pointed out as biomarkers of this metabolism [6,7], their plasma concentrations are subjected to fluctuations as a response to post-prandial metabolism [8,9]. Diurnal variations of glucose and BHB are also observed in adult animals as a response to hormonal regulation and feed intake [10]. Data from Nussio et al. [11] showed that the peak of glucose concentration for calves fed whole milk (WM) occurs 2 hours after feeding, and, therefore, this time has been used to perform blood collections in several trials [12,13,14,15,16,17,18,19,20]. However, some more recent studies from our laboratory have not shown the expected reduction in glucose concentration with increasing age of calves, even though BHB increases, as a function of starter intake increase, are observed. Glucose concentration did not change with age, even with increased starter intake and concentrations of BHB, especially when calves were fed milk replacer (MR), regardless of the liquid diet volume fed [13,15,16,18,19]. These findings differ from what is expected and observed by others [12,14]. 

The different commercial milk replacer compositions, especially due to the presence of plant-based ingredients [21], can affect the metabolism of nutrients and, consequently, the concentrations of some metabolites. The same pattern of glucose concentration was also observed in studies in which whole milk was offered in greater volumes than conventional or restricted liquid-feeding programs [17,20]. Thus, this sampling time may not be the most suitable for animals fed with MR or in a more biological feeding regimen, leading to a misunderstanding of calf metabolism. These data suggest that the glucose peak may occur at a different time point after the liquid diet supply, according to the liquid diet composition. 

Alternatively to evaluating glucose concentrations, fructosamine (FRUC) could be a measure to understand the glycemic status and metabolism of calves with a developing rumen. As the rumen develops, there is an increase in BHB and a decrease in glucose concentrations. However, glucose is highly affected by feed intake, and thus a more independent metabolite could improve the understanding of this process, regardless of feeding and sampling time. The FRUC is a glycosylated protein derived from an irreversible non-enzymatic reaction of glucose with a protein, usually albumin [8]. It is a reliable parameter for assessing glucose metabolism, as it indicates the long-term average glucose concentration but with no diurnal, post-prandial, or glucose concentration effects [22]. Thus, FRUC could eliminate the effect of transient changes due to feeding intake and fasting on blood glucose concentrations, and could be used as a reliable biomarker for rumen development. The normal range of FRU for cattle is between 213 to 265 μmol/L [22], and even though some studies have evaluated FRUC in production animals to understand energy metabolism, only one study was conducted with calves. Coppo [23] reported a decrease in FRU concentrations from 294–303 μmol/L in preweaned calves to 215–232 μmol/L in 6-month-old weaned calves. The decreasing pattern as calves aged was also observed for glucose concentrations, suggesting a good correlation between both metabolites [23].

The aim of this study is to evaluate the age and post-prandial variations in selected metabolites concentration that may indicate a shift in metabolism, according to the liquid diet fed to dairy calves. Additionally, to evaluate the replacement of glucose by fructosamine, determination to better understand the glycemic status and possible use of it as a biomarker of rumen development. We hypothesize that dairy calves fed MR have a delayed glucose peak compared to calves fed WM, suggesting differences in sampling time. In addition, we believe that as the feeding intake affects plasma glucose concentration, a more post-prandial independent biomarker would be informative for dairy calf nutrition research.

## 2. Materials and Methods

### 2.1. Animal, Experimental Design, and Treatments

The study was conducted using 16 newborn Holstein calves (birth weight of 33.7 ± 2.3 kg), immediately separated from their dam at birth, and weighed. The newborns were fed 10% of the birth weight of high-quality colostrum (>50 g/L IgG) in the first six hours of life [24]. Only animals presenting serum protein above 5.5 g/dL at 48 hours of life were enrolled in the study. Navels were treated with a 7% iodine solution for three consecutive days after birth. 

Calves were blocked according to sex, weight, and date of birth, and were randomly assigned to one of the following treatments: Whole milk (WM; *n* = 8); or Milk replacer (MR; Sprayfo Azul, Nutreco Brasil Nutrição Animal LTDA, SP; *n* = 8). The solids of MR were increased to 14% solids to reduce differences in protein and fat intake by calves fed WM. All calves were fed 6 L/d, divided into two feedings at 700 h and 1700 h, in individual open buckets.

### 2.2. Facilities, Intake, and Body Measurements

The calves were individually housed in suspended cages (113 cm × 140 cm) until 14 days of age, after which they were moved to individual wood shelters distributed in a grass field. The shelters were moved daily in order to keep the animals in a clean and dry place. All animals had free access to water and calf starter (Bezerra AgMilk Agroceres Multimix Animal Nutrition Ltd.a., Rio Claro, SP, Brazil). Individual intake of liquid and solid diets was recorded daily until 8 weeks of age, which is when the study ended. Intake of liquid diet lactose and protein was calculated based on liquid diet composition (DM basis) and daily intake averaged by week. Weekly, just before feeding, calves were weighed on a mechanical scale (ISO-300, Coimma Ltd.a., Dracena, SP, Brasil), and heart girth (Bovitec, Sao Paulo, SP, Brazil), wither height, and hip-width (Carci, Sao Paulo, SP, Brazil) were measured. 

### 2.3. Fecal Score and Health

Fecal consistency was scored on a scale of 1 to 4, where the fecal score of 1 = normal consistency, 2 = semi-formed or pasty, 3 = loose feces, and 4 = watery feces according to the method described by Larson et al. [25]. The diarrhea episode was considered when the fecal score was ≥ 3 for more than one day, when calves received an oral rehydration solution (5 g of NaCl, 25 g of dextrose, and 10 g of bicarbonate/L). The rectal temperature was measured daily using a digital thermometer, and fever was considered when the temperature was higher than 39.5 °C. All health problems were recorded and treated according to veterinary recommendations.

### 2.4. Sampling and Laboratory Analysis

Samples of MR and starter were collected monthly for composition analysis (Table 1). Starter samples were analyzed for dry matter (DM), ash, and ether extract (EE), according to the procedures of the Association of Official Analytical Chemists (AOAC method 925.40; method 942.05; method 920.39, respectively). Crude protein (CP) was determined through combustion using FP-528 nitrogen analyzer (Leco Corporation, St. Joseph, MI, USA). Neutral detergent fiber (NDF) was determined according to Van Soest et al. [26]. The NFC of the starter and MR were estimated according to the equation: NFC (%) = 100% − (% NDF + % CP + % Fat + % Ash), according to Mertens [27]. Milk samples were collected weekly from the bulk tank during the overall experiment period and analyzed by a commercial laboratory (Clínica do Leite, São Paulo, Brazil). The average milk composition was: 3.59% protein, 3.95% fat, 4.42% lactose, 12.95% total solids, and somatic cell count (SCC) of 400 × 10³ cells/mL. 

### 2.5. Blood Parameters

Blood samples were taken weekly at 0 h, 1 h, 2 h, 4 h, and 8 h hours after morning feeding until 8 weeks of age to understand the post-prandial effects of different liquid diets on selected metabolites. Blood samples were collected via puncture of the jugular vein in vacuum tubes containing sodium fluoride as an antiglycolytic and potassium EDTA as an anticoagulant to obtain plasma for glucose, fructosamine, and BHB analysis. Another tube with a clot activator was used to obtain serum for total protein and albumin analysis. Samples were centrifuged at 2000× *g* for 25 min at 4 °C to obtain serum or plasma, which were stored in plastic tubes and a freezer (−10 °C) for subsequent analysis. Commercial enzymatic kits from LABTEST Diagnóstica S.A. (Lagoa Santa, MG, Brazil) were used to analyze glucose (Ref.: 133.1/500), fructosamine (Ref.: 97-6/15), total serum protein (Ref. 99), and albumin (Ref. 19-1/250); and from RANDOX Laboratories – Life Science Ltd. (Crumlin, UK) to analyze β-hydroxybutyrate (Ref.: RB1007) in an Automatic System for Biochemistry-Model SBA-200 (CELM, Barueri, SP, Brazil). Another blood sample was collected in a tube with K3 EDTA to evaluate hematocrit, and an aliquot was used for capillary hematocrit using a microhematocrit centrifuge at 2000× *g* for 10 min (model SPIN 1000, Microspin, Sao Paulo, Brazil).

### 2.6. Statistical Analyses

The performance, fecal scores, and blood parameters were analyzed as repeated measures over time using the PROC MIXED of the SAS package (version 5.0, SAS Institute Inc., Cary, NC, USA), according to the model: Yijk = μ + Ti + Bj + Wk + TWik + Eijk. Where Yijk = response variable; μ = overall mean, Ti = liquid diet effect (WM or MR); Bj = random block effect; Wk = age effect (hour or week of age); TWik = was the effect of the interaction of liquid diet and hour or age; Eijk = was the residual effect. The non-repeating variables were evaluated using the statistical model: Yij = μ + Ti + bj + eij where μ = overall mean; Ti = liquid diet effect (WM or MR); bj = random block effect; eij = residual effect. The treatment means were compared using the Tukey-Kramer adjustment test. Significance was declared at *p* ≤ 0.05 and tendency at 0.05 > *p* > 0.10. 

## 3. Results

**Figure 1:**
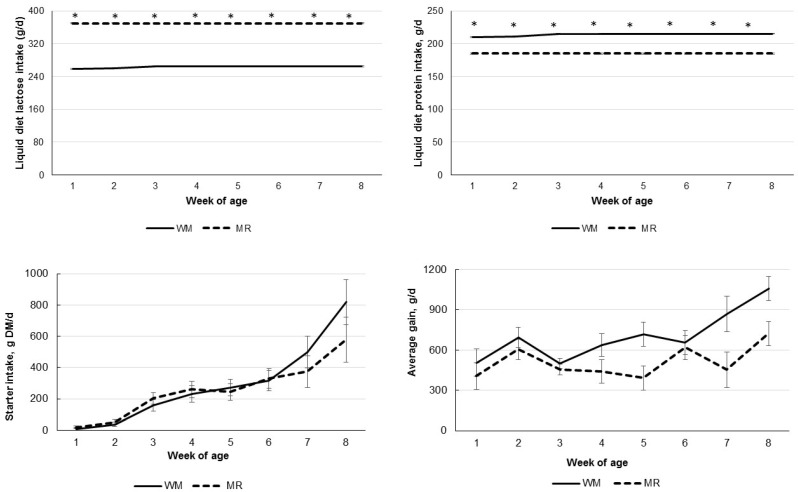
Liquid diet lactose and protein intake, starter intake, and average daily gain according to the age of calves fed whole milk (WM) or milk replacer (MR) as their liquid diet. * Denotes difference between liquid diet with *p* < 0.05.

**Figure 2:**
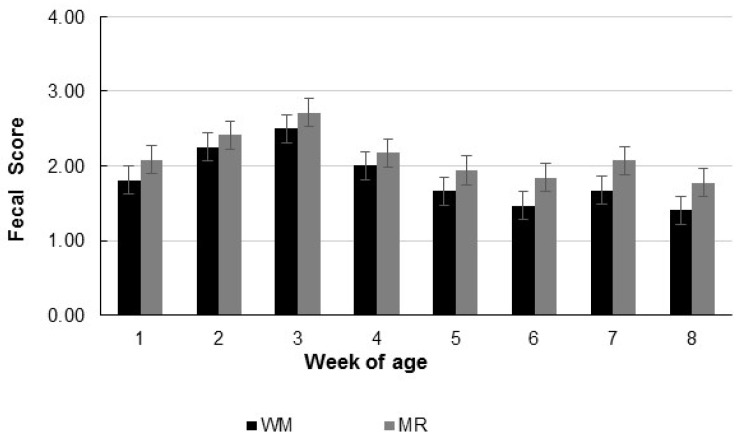
Fecal scores according to the age of calves fed whole milk (WM) or milk replacer (MR) as liquid diet.

Total dry matter intake (TDMI) was not affected by the liquid diets; however, it increased with the age of the calves. Calves fed MR presented a higher liquid diet intake (*p* < 0.001), representing almost 80% of TDMI. Due to differences in liquid diet composition, MR-fed calves presented higher liquid diet lactose intake (*p* < 0.001) but lower liquid diet protein intake (*p* < 0.001). Those intakes were also affected by age and the interaction of liquid diet and age(*p* < 0.02), with differences in both lactose and protein liquid diet intake in all evaluated weeks (Figure 1). On the other hand, the starter concentrate intake was not affected by liquid diets or their interaction with age (Table 2; Figure 1); however, it increased with the calves’ age. The average daily gain (ADG) increased with age (*p* = 0.01; Table 2 and Figure 1) and was higher for calves fed WM (*p* < 0.01), resulting in higher final weight for those calves (*p* = 0.02; Table 2). The gain in body measurements evaluated per week was also higher for calves fed WM (Table 2; *p* < 0.05) but were not affected by age or the interaction of liquid diet and age. Fecal scores were higher for MR-fed calves (*p* < 0.03), suggesting more fluid feces. However, fecal scores have shown an age effect, increasing values until the third week of age and decreasing after that (Figure 2).

**Figure 3:**
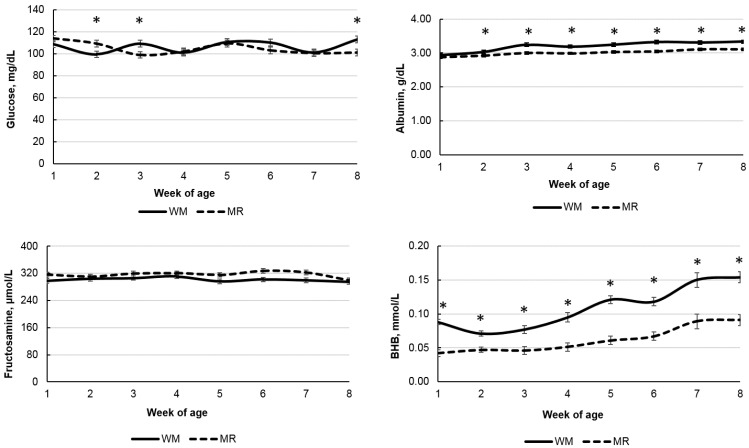
Blood metabolites according to the age of calves fed whole milk (WM) or milk replacer (MR) as liquid diet. * Denotes difference between liquid diets with *p* < 0.05.

**Figure 4:**
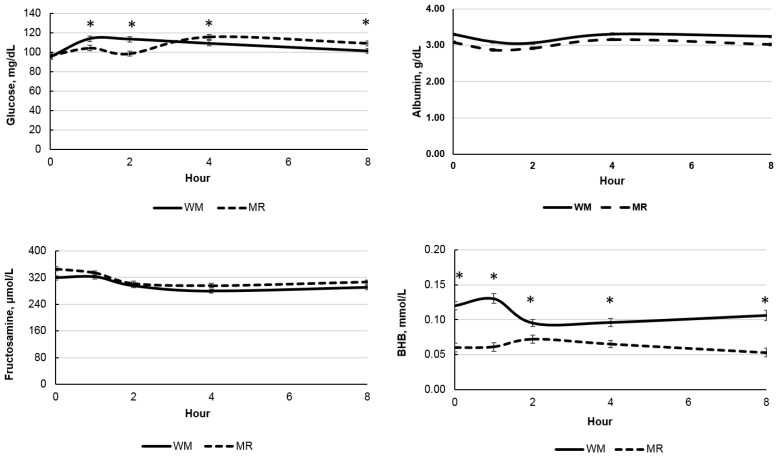
Blood metabolites according to the sampling time of calves fed whole milk (WM) or milk replacer (MR) as liquid diet. * Denotes difference between liquid diet with *p* < 0.05.

All evaluated metabolites were affected by age (Table 3; *p* < 0.001) and by sampling time (hour after liquid diet feeding; *p* < 0.001). However, there was no expressive reduction in glucose concentration as calves aged, while BHB increased as expected (Figure 3). The concentration of BHB was higher (*p* < 0.01) for WM-fed calves than MR-fed calves in all evaluated weeks of age (Figure 3), while albumin concentrations were higher from week 2 until the end of the evaluation period. There was a tendency for FRU to be lower and total protein to be higher for WM-fed calves (*p* < 0.06; Table 3). Glucose (*p* < 0.01), BHB (*p* < 0.001), and albumin concentrations were affected by the liquid diet and age interaction and by the liquid diet and sampling time interaction (Table 3; Figure 3 and Figure 4). Glucose concentrations were higher for MR-fed calves at week 2 but lower at weeks 3 and 8 than those fed WM. While the peak of glucose concentration occurred at 1 and 2 h hours after feeding for calves fed WM, it occurred only at 4 h after feeding for calves fed MR (Figure 4). BHB was higher for WM-fed calves in all evaluated sampling times, while albumin and FRUC were unaffected by the interaction of liquid diet and sampling time.

## 4. Discussion

Calves fed with MR had lower performances compared to those fed whole milk. Since starter intake was not affected by the liquid diet, the lower quality of MR compared to WM concerning protein, fat, and lactose levels and quality may explain this result. The increase in MR solids to 14% was insufficient to result in a similar ADG to that observed for WM-fed calves. Indeed, calves fed MR had a higher intake of lactose; however, a lower intake of protein, which, together with the lower quality of the MR protein source, may have impaired growth. The balance between metabolizable energy and protein is mandatory for higher growth rates [28]. The milk replacer composition can influence the performance of dairy calves, as shown in different studies [29,30,31].

Nevertheless, this could be a problem, as it can affect the intestinal bacterial community composition [32], resulting in an abundance of pathogenic microorganisms in periods of diarrhea in the first weeks of life [33], which can compromise the performance of these animals, and consequently affect the metabolic pattern. The meta-analysis study by Quigley et al. [34] concluded that the nutrient digestibility of the liquid diet (DM, N, and fat) is maximum up to approximately 30 days. In this manner, the liquid diet composition that may cause more intestinal disturbances during early life can affect nutrient absorption and metabolism and, consequently, the performance of these animals. However, despite differences in fecal scores according to the liquid diet, the higher scores for MR calves may not be considered diarrhea. Liquid diet affected growth since body measurements gain was also higher for calves fed WM.

Observed glucose concentration values are within those reported in the literature, where healthy calves normally have a plasma glucose concentration near 100 mg/dL [12,35]. The plasma glucose concentration showed an effect of age, with a slight drop in concentrations over the evaluation period. This reduction occurs since, with the gradual metabolic transition from pre-ruminant to a functional ruminant, the animal no longer uses glucose from lactose absorbed by the small intestine as its main energy source and starts using SCFAs from the fermentation of the initial concentrate as a source of energy [36,37]. However, the drop in glucose concentrations between the first and third weeks of age in animals fed with MR and between the first and second week for animals fed with WM may have occurred as a result of the higher occurrence of diarrhea in the respective periods, which may have reduced glucose absorption and availability. 

Calves were fed 6 L/d of MR or WM, and even though lactose intake was higher for calves fed MR, that had a minor impact on glucose concentrations as calves aged. Considering the increases in starter intake and the age effect on BHB, glucose concentrations were expected to decrease with the increasing age of calves, as observed by others [2,12,14]. 

Glucose peak concentration occurred earlier for WM (1 and 2 h after feeding) than for MR (4 h after feeding), which was unexpected since MR had a higher lactose concentration. However, lactose absorption and use as an energy source may have been affected by the coagulation of WM casein in the abomasum, which normally does not occur with MR because it lacks casein [38]. Nevertheless, most research on the effect of liquid diet clotting has only looked at effects on crude protein digestibility and diarrhea occurrence and performance [39,40]. Through the action of renin, pepsin, and the acidic environment, a clot composed of proteins and fats is formed, and the whey composed of water, minerals, lactose, and other proteins passes into the duodenum, and is quickly absorbed and digested. Thus, the MR does not form a clot and passes directly to the duodenum, where it will be digested and absorbed more slowly, which could delay the glucose peak, as observed in the present study.

On the other hand, FRU is a metabolite with no post-prandial effects [22], meaning feeding should not alter its concentration according to time. Nevertheless, that was observed in the present study. Fructosamine concentrations are believed to be proportional to mean glucose concentrations for 2 to 3 weeks [41] and can indicate the average glucose concentration to which serum proteins have been exposed for a long period [42]. Therefore, FRU should be more stable than glucose and could be a better predictor of the glycemic status and rumen development as it is not affected by the collection time in relation to the feeding supply. That could improve the understanding of the metabolic shift of calves as regards the rumen development process. As mentioned earlier, several studies fail to show a decrease in glucose concentrations as calves increase starter intake and BHB concentrations [13,15,16,18,19], both good indicators of rumen development. We have hypothesized that this could occur in response to post-prandial effects or levels of liquid diet fed. Fructosamine concentrations were higher than the normal range for adult cattle (213.4 µmol/L to 265 µmol/L) as described by Jensen et al. [22]; but similar to values observed by Coppo [23] for preweaning calves at (294–303 µmol/L). Once glucose concentrations are high, there is also an increase in fructosamine levels, as demonstrated by Coppo [23] in studies with dairy calves. In the present study, there was a reduction in the concentrations of fructosamine, as expected for animals with reduced glucose as a function of ruminal development. Coppo [23] also observed a decreased FRU and glucose concentration as calves aged, which negatively correlated to growth. However, the reduction in FRUC was not as clear as we expected, but since it reflects glucose concentration of about 2 to 3 weeks before [43], an 8 week evaluation period potentially may not have been enough to observe that. Unfortunately, we could only follow calves after the 8th week, when calves were still receiving the liquid diet, and that could have limited our understanding of FRUC dynamics and correlation with glucose concentration after weaning. However, the small decrease as calves aged suggests that this metabolite does not reflect the transition from the pre-ruminant to a functional ruminant metabolism as well as BHB.

Nevertheless, since this glycated protein should be independent of the glycemic status at the time of collection, this effect was not expected. According to Jensen et al. [22], the FRUC concentration results from its synthesis and elimination rate. The first is regulated by the protein concentration and composition and glucose concentration according to the lifetime of several proteins. The second reflects the protein turnover rate, mainly that of albumin. Indeed, albumin concentrations were lower for calves fed MR, which presented higher FRU. We also speculate that there may be an effect related to hemoconcentration as a function of liquid diet consumption. 

The concentrations of BHB were higher for animals fed with WM, suggesting a superior ruminal development than for MR-fed calves, even with no difference in the average starter intake. The increase in BHB concentrations with the advancing age of the animals was similar for both liquid diets, and occurred in response to the increase in starter concentrate intake and consequent rumen development. However, BHB was also affected by sampling time with higher concentrations for WM-fed calves throughout the 8 h evaluating period. Senn et al. [9] have also observed increases in BHB after a meal, mainly in early ages, when calves were not yet considered a functional ruminant, suggesting a high activity of β-oxidation hepatic enzymes. On the other hand, BHB concentrations may also be affected by stress and feed intake in transition cows [44]. Thus, both rumen and hepatic-originated BHB may affect its plasma concentration during the day.

## 5. Conclusions

The supply of milk replacer resulted in lower performance of dairy calves, even using a dilution rate of 14% solids. The peak of plasma glucose concentration was observed 1 h and 4 h after the supply of WM or MR, respectively. Thus, these would be the best collection times to assess glycemic status according to the liquid diet fed to dairy calves. However, fructosamine did not prove to be an informative metabolite for an indirect evaluation of a metabolic shift from pre to a functional ruminant because of the sampling time effect and the small reduction as a function of age, even though BHB concentrations increased. However, a longer evaluation period is needed to improve the understanding of fructosamine, glucose, and BHB as indicators of rumen development in fully weaned calves.

## Figures and Tables

**Table 1:** Chemical composition of calf starter and milk replacer.

KERRYPNX	Calf Starter ^1^	Milk Replacer ^2^
Dry Matter, %	87.4	96.5
Total Solids, %	-	14
Ash, %DM	8.6	8.7
Crude Protein, %DM	25.4	22.9
Ether Extract, %DM	5.9	18.8
NDF ^3^, %DM	14.7	1.1
NFC ^4^, %DM	45.4	48.5
Lactose, %DM	-	44.0

^1^ Commercial calf starter (Ração Bezerra AgMilk Agroceres Multimix Nutrição Animal Ltd.a., Rio Claro, SP, Brazil). ^2^ Commerical milk replacer (Sprayfo Azul, Nutreco Brasil Nutrição Animal LTDA, SP, Brazil) diluted to 14% solids. ^3^ NDF, Neutral detergent fiber. ^4^ NFC, Non-fiber carbohydrate.

**Table 2:** Intake, performance, and body measurements of dairy calves fed whole milk (WM) or milk replacer (MR).

Item	Liquid Diet ^1^		SEM ^2^	*p*-Value ^3^		
	WM	MR		T	A	T × A
**Intake, g DM/d**						
Total	1028	1065	50.53	0.60	<0.001	0.49
Liquid diet	772.5	840.0	2.08	<0.001	0.02	0.02
Liquid diet lactose	263.4	369.6	0.708	<0.001	0.02	0.02
Liquid diet protein	213.9	184.8	0.57	<0.001	0.02	0.02
Solid diet	255.6	225.2	37.53	0.574	<0.001	0.76
**Average gain, g/d**	705.1	512.5	27.1	<0.01	0.01	0.24
**Body weight, kg**						
Initial	33.30	34.13	2.366	0.80	-	-
Final	72.79	62.29	2.786	0.02	-	-
**Body measurements gain, cm/week**						
Wither height	1.87	1.47	0.098	<0.01	0.73	0.57
Hip width	0.74	0.53	0.050	0.02	0.35	0.91
Heart girth	2.94	2.41	0.270	0.05	0.33	0.61
**Fecal score**	1.8	2.1	0.08	0.03	<0.01	0.99

^1^ WM: whole milk; MR: milk replacer; ^2^ Standard error of the mean; ^3^ T: Liquid diet effect; A: age effect; T × A: Liquid diet and age interaction effect.

**Table 3:** Blood metabolites of dairy calves fed whole milk (WM) or milk replacer (MR).

	Liquid Diet ^1^		SEM ^2^	*p*-Value ^3^				
	WM	MR		T	A	H	T × A	T × H
Glucose, mg/dL	106.56	104.68	2.08	0.36	<0.01	<0.01	<0.01	0.01
BHB, mmol/L	0.110	0.062	0.005	<0.01	<0.01	<0.01	<0.01	0.01
Fructosamine, μmol/L	301.60	316.59	5.13	0.06	<0.01	<0.01	0.18	0.19
Total protein, g/dL	6.34	5.88	0.16	0.07	<0.01	<0.01	0.97	0.22
Albumin, g/dL	3.20	3.01	0.02	<0.01	<0.01	<0.01	<0.01	0.07

^1^ WM: whole milk; MR: milk replacer; ^2^ Standard error of the mean; ^3^ T: Liquid diet effect; A: age effect; H: sampling hour effect; T × A: Liquid diet and age interaction effect; T × H: Liquid diet and sampling hour interaction effect.

## Data Availability

The data presented in this study are available on request from the corresponding author. The data are not publicly available due to restrictions by the research group.
